# Higher BMI reduces mortality in elderly and Stage III colorectal cancer patients: insights from a multicenter cohort study

**DOI:** 10.3389/fnut.2025.1655707

**Published:** 2025-09-19

**Authors:** Yihuan Qiao, Boyu Kang, Yu Jiang, Zecheng Zhang, Baoliang Hu, Jiawei Song, Hongjiang Ma, Shuai Liu, Yongtao Du, Qi Wang, Yajie Guo, Shihao Qin, Zhaobang Tan, Jun Zhu, Yi Huang, Jipeng Li

**Affiliations:** ^1^Department of Digestive Surgery, Xijing Hospital of Digestive Diseases, The Fourth Military Medical University, Xi'an, China; ^2^State Key Laboratory of Holistic Integrative Management of Gastrointestinal Cancers and National Clinical Research Center for Digestive Diseases, Xijing Hospital of Digestive Diseases, Fourth Military Medical University, Xi'an, China; ^3^Department of General Surgery, Chinese People's Liberation Army Central Theater Command General Hospital, Wuhan, China; ^4^School of Clinical Medicine, Yan'an University, Yan'an, China; ^5^School of Clinical Medicine, Xi'an Medical University, Xi'an, China; ^6^Department of General Surgery, The Southern Theater Air Force Hospital, Guangzhou, China; ^7^Department of Preventive Health Care, The First Affiliated Hospital of Air Force Medical University, Xi'an, China; ^8^Department of Experiment Surgery, The First Affiliated Hospital of Air Force Medical University, Xi'an, China

**Keywords:** colorectal cancer, BMI, age, TNM stage, obesity paradox, mortality risk

## Abstract

**Background:**

Colorectal cancer (CRC) is a major global health concern, with obesity rates rising and an observed obesity paradox where higher body mass index (BMI) is linked to better outcomes in certain patient groups. This study aims to explore how age and tumor stage modify the association between BMI and mortality risk in CRC patients.

**Materials and methods:**

This retrospective cohort study included 4,114 CRC patients who underwent surgery between December 2013 and December 2019. Patients were categorized by BMI, age, and TNM stage. Multivariate Cox regression models and Kaplan-Meier survival analyses were used to assess the impact of BMI on mortality risk, adjusting for potential confounders such as age, sex, and cancer stage.

**Results:**

Higher BMI was associated with lower mortality risk across the study population. Specifically, the protective effect of higher BMI was most pronounced in patients aged 65 and older and in those with Stage III disease. The multivariate Cox regression analysis revealed that each unit increase in BMI was associated with a 7% decrease in mortality risk. The Kaplan-Meier survival analysis showed significant survival benefits for higher BMI in patients aged 65 and older and in Stage III patients.

**Conclusions:**

Higher BMI is associated with lower mortality risk in colorectal cancer patients, particularly in those aged 65 and older and those with Stage III disease. These findings highlight the importance of considering BMI, age, and TNM stage jointly in clinical practice for CRC patients.

## 1 Introduction

Colorectal cancer (CRC) is the third most diagnosed cancer worldwide and the second leading cause of cancer-related deaths (1). With the improvement in living standards, the incidence of obesity has significantly increased, leading to a higher number of obese patients diagnosed with colorectal cancer (2, 3). The relationship between obesity and colorectal cancer is complex and paradoxical (4). While obesity is a known risk factor for the development of CRC, recent studies have shown that higher BMI may be associated with better outcomes in certain patient populations, a phenomenon known as the “obesity paradox” (5–7).

Numerous studies have investigated the relationship between BMI and patient prognosis in colorectal cancer, but consensus has not been reached (8). Some studies suggest that higher BMI is associated with increased mortality risk, while others indicate a protective effect of higher BMI on survival outcomes (3, 4, 9, 10). Age and tumor stage are important factors that can influence the relationship between BMI and patient prognosis (4, 11, 12). However, few studies have comprehensively explored how age and tumor stage modify the association between BMI and mortality risk in colorectal cancer patients.

Therefore, this study aims to investigate the age- and stage- modified associations between BMI and the mortality risk in CRC. By conducting a retrospective cohort study, we will analyze the combined effects of BMI, age, and TNM stage on mortality risk. This research will provide valuable insights for developing more personalized treatment strategies and improving the prognosis of colorectal cancer patients.

## 2 Materials and methods

### 2.1 Study design and participants

This retrospective cohort study included colorectal cancer patients who underwent surgery at the Department of Digestive Surgery, First Affiliated Hospital of the Air Force Medical University, and the Department of General Surgery, Shaanxi Provincial People's Hospital, from December 2013 to December 2019. Inclusion criteria: (1) Pathologically diagnosed with colorectal adenocarcinoma, staged T1-4a (pT1-4a), N0/N+, M0 per the American Joint Committee on Cancer (AJCC) Eighth Edition Cancer Staging Manual; (2) Underwent radical surgery (R0 resection); (3) Had complete clinical and pathological data; (4) Had complete follow-up information; (5) Post-operative adjuvant chemotherapy was administered according to the NCCN guidelines: patients with Stage II disease plus high-risk features and all patients with Stage III disease were offered adjuvant therapy. Exclusion criteria: (1) Other concurrent malignancies; (2) Preoperative chemoradiotherapy; (3) Metabolic diseases (hypothyroidism, Cushing's syndrome, polycystic ovary syndrome, diabetes, or metabolic syndrome); (4) Long-term use of glucocorticoids, insulin, or GLP-1 receptor agonists. This study complies with the Declaration of Helsinki and was conducted in accordance with the protocol approved by the ethics committee of the First Affiliated Hospital of the Air Force Military Medical University, with the ethics protocol number: KY20232232-F-1. All participants provided informed consent and were informed about the study's purpose and data confidentiality.

### 2.2 Clinical data collection

We collected patients' baseline and pathological data. Baseline data included gender, age, and BMI at diagnosis. Age groups were Early-onset (< 50), Intermediate-onset (50 ≤ age < 64), and Late-onset (≥65) (13). BMI categories were underweight (< 18.5), normal (18.5–24.9), overweight (25–29.9), and obese (≥30) per WHO criteria. Pathological data included tumor location, differentiation degree (well, moderately, poorly), DNA mismatch repair status (dMMR/pMMR), vascular invasion (negative/positive), number of harvested lymph nodes, and TNM stage (I, II, III). Tumor locations were right-sided colon cancer (cecum to hepatic flexure), left-sided colon cancer (splenic flexure to rectosigmoid junction), and rectal cancer. Follow-up was conducted via outpatient visits and phone calls. Patients had follow-ups every 3 months for the first 2 years, then every 6 months thereafter. Overall survival (OS) was calculated from the date of initial surgery to death or last follow-up. The primary study endpoint was overall survival, and the censoring date for follow-up was 30 June 2024.

### 2.3 Statistical analysis

Quantitative data following a normal distribution are presented as the mean ± standard deviation (x ± s), with intergroup comparisons conducted using the independent samples *t*-test. For quantitative data with a skewed distribution, the median (Q1, Q3) is used, and intergroup comparisons are performed using the Mann-Whitney U test. Categorical data are described using absolute numbers and percentages, with intergroup comparisons made via the chi-square test. To assess the overall impact of BMI on mortality risk, hazard ratio (HR) curves are plotted for the entire study population. Additionally, HR curves are separately plotted for different TNM stages and age groups to compare the differential effects of BMI on mortality risk. The Kaplan-Meier method was employed to estimate survival probabilities, and log-rank tests were used to compare survival differences across groups. Cox proportional hazards regression models were utilized to assess the relationship between BMI and mortality risk, with adjustments for potential confounding variables such as age, sex, and cancer stage. Stratified survival analyses were conducted to explore variations in the impact of BMI on mortality risk across different age and stage subgroups. All analyses were performed using R 4.2.1 and SPSS 24.0. *P*-values were only calculated for the primary hypotheses (e.g., associations between BMI, age, stage, and mortality risk in Cox models). No *p*-values were reported for baseline characteristic comparisons to avoid multiplicity issues. A two-tailed *p*-value < 0.05 was considered statistically significant.

## 3 Results

### 3.1 Demographic characteristics

**Figure 1:**
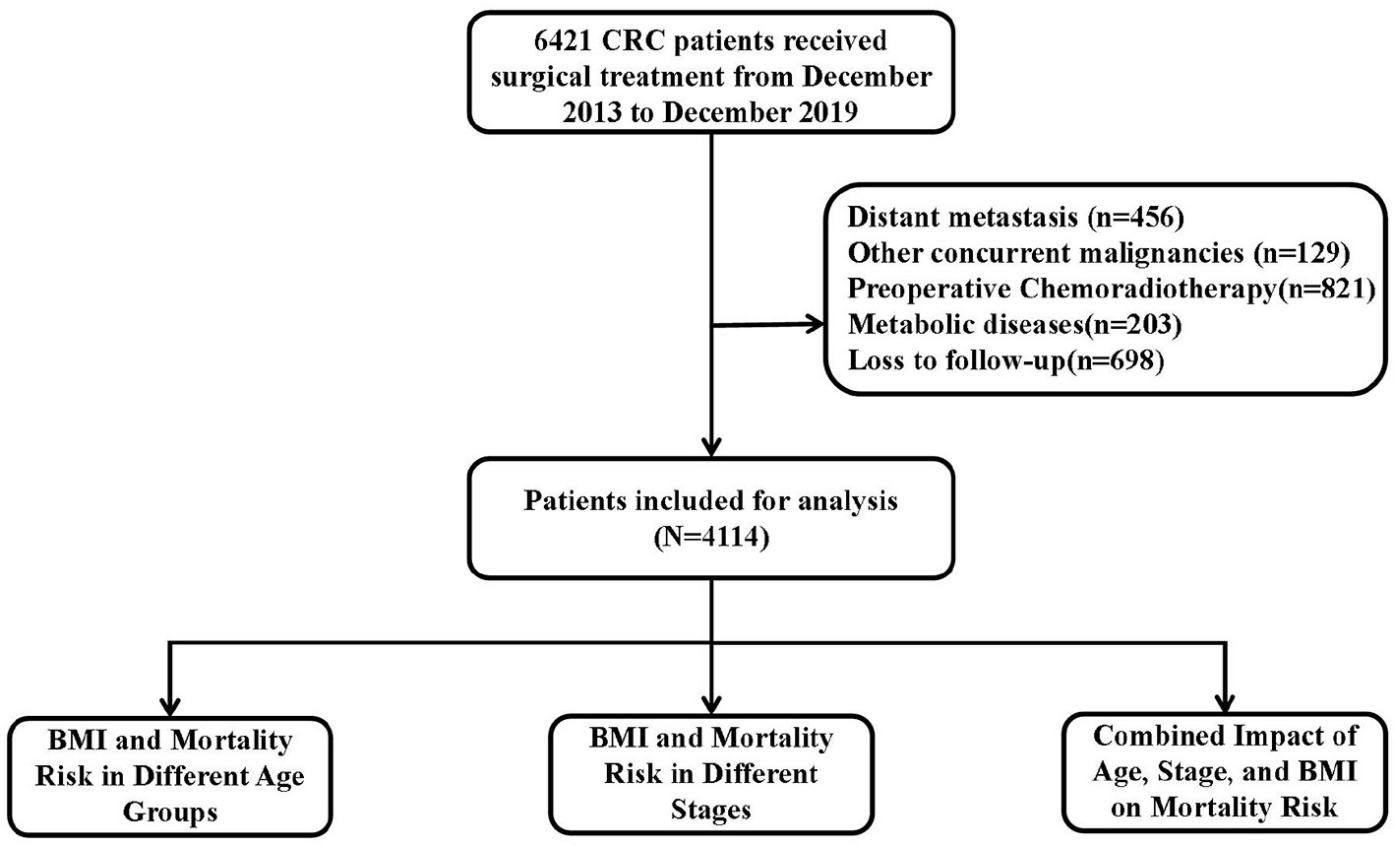
Flowchart of the study.

This study enrolled 6,421 CRC patients confirmed by pathology, 2,307 patients were excluded for the following reasons: distant metastasis (*n* = 456), other concurrent malignancies (*n* = 129), preoperative chemoradiotherapy (*n* = 821), metabolic diseases (*n* = 203), and loss to follow-up (*n* = 698), and 4,114 were finally analyzed after screening. Figure 1 shows the study flowchart. Among the included patients, 59.6% were male and 40.4% were female; 22.8% had right-colon cancer, 24.9% left-colon cancer, and 53.3% rectal cancer. The mean age at diagnosis was 61.1 years (SD = 12.1). The proportions of patients in stages I, II, and III were 18.3%, 37.9%, and 43.8%, respectively. The follow-up time in our study ranged from 2 to 95 months, with a median follow-up of 42 months. We categorized patients based on BMI, age, and stage and analyzed the baseline characteristics of each group.

BMI stratification showed a progressive increase in male representation from underweight (46.6%) to obese categories (68.4%), with generally similar distributions of other variables (Table 1). In the baseline characteristic analysis by age groups, younger patients (< 50 years) had lower median BMI (22.0 kg/m^2^), larger tumors (median 4.0 cm), higher dMMR prevalence (17%), and more lymph node involvement (median 1 positive node) compared to older groups, while elderly patients (≥65 years) had fewer harvested lymph nodes (median 17 vs. 18 in < 50 y; Table 2). Stage stratification demonstrated Stage III patients had substantially higher rates of vascular invasion (77.5% vs. 34–35% in early stages) and poor differentiation (24.0% vs. 5.9–15.2%), with decreasing rectal cancer prevalence from Stage I (65.5%) to Stage II (43.5%; Table 3).

**Table 1:** Baseline characteristics of BMI groups.

**Characteristics**	**Underweight**	**Normal**	**Overweight**	**Obese**
	**221**	**2,901**	**897**	**95**
Age, year, median (IQR)	64 (54, 73)	62 (53, 69)	62 (54, 69)	62 (53, 69)
**Gender**, ***n*** **(%)**				
Male	103 (46.6%)	1,687 (58.2%)	599 (66.8%)	65 (68.4%)
Female	118 (53.4%)	1,214 (41.8%)	298 (33.2%)	30 (31.6%)
Size, cm, median (IQR)	4 (3, 5)	4 (3, 5)	4 (3, 5)	4 (3, 5.5)
Positive lymph nodes, median (IQR)	0 (0, 1)	0 (0, 1)	0 (0, 0)	0 (0, 0)
Lymph nodes harvest, median (IQR)	17 (15, 21)	17 (15, 20)	17 (15, 20)	17 (15, 20)
Ki67, %, median (IQR)	80 (60, 80)	70 (60, 80)	70 (60, 80)	70 (60, 80)
**TNM stage**, ***n*** **(%)**				
I	31 (14.1%)	540 (18.6%)	170 (19.0%)	12 (12.6%)
II	92 (41.6%)	1,090 (37.6%)	333 (37.1%)	44 (46.3%)
III	98 (44.3%)	1,271 (43.8%)	394 (43.9%)	39 (41.1%)
**DNA mismatch repair**, ***n*** **(%)**				
dMMR	26 (11.8%)	314 (10.8%)	80 (8.9%)	8 (8.4%)
pMMR	195 (88.2%)	2,587 (89.2%)	817 (91.1%)	87 (91.6%)
**Perineural/vascular invasion**, ***n*** **(%)**				
Positive	117 (52.9%)	1,516 (52.3%)	503 (56.1%)	57 (60.0%)
Negative	104 (47.1%)	1,385 (47.7%)	394 (43.9%)	38 (40.0%)
**Differentiation**, ***n*** **(%)**				
Well-differentiated	24 (10.9%)	260 (9.0%)	77 (8.6%)	4 (4.2%)
Moderately-differentiated	159 (71.9%)	2,139 (73.7%)	665 (74.1%)	73 (76.8%)
Poorly-differentiated	38 (17.2%)	502 (17.3%)	155 (17.3%)	18 (19.0%)
**Site**, ***n*** **(%)**				
Left-sided colon	55 (24.9%)	704 (24.3%)	234 (26.1%)	29 (30.5%)
Right-sided colon	60 (27.1%)	675 (23.3%)	193 (21.5%)	14 (14.8%)
Rectum	106 (48.0%)	1,522 (52.4%)	470 (52.4%)	52 (54.7%)

IQR, interquartile range; BMI, Body mass index; TNM, Tumor-Node-Metastasis Staging System. Baseline characteristics are presented descriptively as median (IQR) or n (%). No p-values are reported to avoid the Table 2 fallacy, as these comparisons do not test the primary hypotheses of this study.

**Table 2:** Baseline characteristics of age groups.

**Characteristics**	**Age ≤ 50**	**50 ≤ Age < 64**	**Age ≥65**
	**676**	**1,753**	**1,685**
BMI, kg/m^2^, median (IQR)	22 (20, 24.7)	22.5 (20, 24.9)	22.4 (20, 24.9)
**Gender**, ***n*** **(%)**			
Male	393 (58.1%)	1,039 (59.3%)	1,022 (60.7%)
Female	283 (41.9%)	714 (40.7%)	663 (39.3%)
Size, cm, median (IQR)	4 (3, 5.8)	4 (3, 5)	4 (3, 5)
Positive lymph nodes, median (IQR)	0 (0, 1)	0 (0, 1)	0 (0, 1)
Lymph nodes harvest, median (IQR)	18 (16, 22)	17 (15, 20)	17 (14, 19)
Ki67, %, median (IQR)	70 (60, 80)	70 (60, 80)	70 (60, 80)
**TNM stage, n (%)**			
I	96 (14%)	367 (21%)	290 (17%)
II	252 (37%)	642 (37%)	665 (39%)
III	328 (49%)	744 (42%)	730 (43%)
**DNA mismatch repair**, ***n*** **(%)**			
dMMR	115 (17%)	173 (9.9%)	140 (8.3%)
pMMR	561 (83%)	1,580 (90%)	1,545 (92%)
**Perineural/vascular invasion**, ***n*** **(%)**			
Positive	394 (58%)	910 (52%)	889 (53%)
Negative	282 (42%)	843(48%)	796 (47%)
**Differentiation**, ***n*** **(%)**			
Well-differentiated	72 (11%)	165 (9.4%)	128 (7.6%)
Moderately-differentiated	450 (67%)	1,312 (75%)	1,274 (76%)
Poorly-differentiated	154 (23%)	276 (16%)	283 (17%)
**Site**, ***n*** **(%)**			
Left-sided colon	173 (26%)	435 (25%)	414 (25%)
Right-sided colon	170 (25%)	383 (22%)	389 (23%)
Rectum	333 (49%)	935 (53%)	882 (52%)

IQR, interquartile range; BMI, Body mass index; TNM, Tumor-Node-Metastasis Staging System. Baseline characteristics are presented descriptively as median (IQR) or n (%). No p-values are reported to avoid the Table 2 fallacy, as these comparisons do not test the primary hypotheses of this study.

**Table 3:** Baseline characteristics of stage groups.

**Characteristics**	**Stage I**	**Stage II**	**Stage III**
	**753**	**1,559**	**1,802**
BMI, kg/m^2^, median (IQR)	22.6 (20, 24.9)	22.5 (20, 24.9)	22.3 (20, 24.8)
Age, year, median (IQR)	61 (54, 68)	63 (53, 70)	61 (53, 69)
**Gender**, ***n*** **(%)**			
Male	431 (57.2%)	983 (61.1%)	1,040 (57.7%)
Female	322 (42.8%)	576 (36.9%)	762 (42.3%)
Size, cm, median (IQR)	3 (2, 4.5)	4 (3, 5)	4.5 (3.5, 6)
Lymph nodes harvest, median (IQR)	16 (14, 19)	18 (15, 21)	17 (14, 19)
Ki67, %, median (IQR)	70 (60, 80)	70 (60, 80)	80 (60, 80)
**DNA mismatch repair**, ***n*** **(%)**			
dMMR	56 (7.4%)	268 (17.2%)	104 (5.8%)
pMMR	697 (92.6%)	1,291 (82.8%)	1,698 (94.2%)
**Perineural/vascular invasion, n (%)**			
Positive	267 (35.5%)	530 (34.0%)	1,396 (77.5%)
Negative	486 (64.5%)	1,029 (66.0%)	406 (22.5%)
**Differentiation**, ***n*** **(%)**			
Well-differentiated	89 (11.8%)	130 (8.3%)	146 (8.1%)
Moderately-differentiated	620 (82.3%)	1,192 (76.5%)	1,224 (67.9%)
Poorly-differentiated	44 (5.9%)	237 (15.2%)	432 (24.0%)
**Site, n (%)**			
Left-sided colon	152 (20.2%)	425 (27.3%)	445 (24.7%)
Right-sided colon	108 (14.3%)	455 (29.2%)	379 (21.0%)
Rectum	493 (65.5%)	679 (43.5%)	978 (54.3%)

IQR, interquartile range; BMI, Body mass index; TNM, Tumor-Node-Metastasis Staging System. Baseline characteristics are presented descriptively as median (IQR) or n (%). No p-values are reported to avoid the Table 2 fallacy, as these comparisons do not test the primary hypotheses of this study.

### 3.2 Multivariate Cox regression analysis

A multivariate Cox regression analysis was performed to assess the relationships between key prognostic factors and mortality risk, adjusting for potential confounders. Each unit increase in BMI was associated with a 7% decrease in mortality risk (HR = 0.933, 95% CI: 0.911–0.956, *P* < 0.001). Mortality risk increased by 2.5% per year of age (HR = 1.025, 95% CI: 1.018–1.031, *P* < 0.001). Tumor size was linked to mortality risk, with a 7.2% increase per cm (HR = 1.072, 95% CI: 1.030–1.116, *P* = 0.001). Each additional positive lymph node was associated with an 11% increase in mortality risk (HR = 1.110, 95% CI: 1.084–1.136, *P* < 0.001). Conversely, each additional harvested lymph node was associated with a 2.4% decrease in mortality risk (HR = 0.976, 95% CI: 0.961–0.991, *P* = 0.002). Patients with dMMR had a 32% lower mortality risk than those with pMMR (HR = 0.680, 95% CI: 0.507–0.912, *P* = 0.01). Compared to stage I, stage III CRC was associated with an 80.3% increase in mortality risk (HR = 1.803, 95% CI: 1.378–2.359, *P* < 0.001). Poorly differentiated tumors carried a 23.1% higher mortality risk than well-differentiated ones (HR = 1.231, 95% CI: 1.024–1.476, *P* < 0.001). Other variables, including gender, S100, CD34, and tumor location, were not significantly associated with mortality risk (Table 4).

**Table 4:** Multivariate Cox regression analysis.

**Characteristics**	**β**	**s_x_**	**Wald**	**HR (95% CI)**	***P*-value**
BMI	−0.069	0.012	31.616	0.933 (0.911–0.956)	< 0.001
Age	0.024	0.003	59.623	1.025 (1.018–1.031)	< 0.001
Size	0.07	0.021	11.509	1.072 (1.030–1.116)	0.001
Positive lymph nodes	0.104	0.012	75.098	1.110 (1.084–1.136)	< 0.001
Lymph nodes harvest	−0.024	0.008	9.591	0.976 (0.961–0.991)	0.002
Gender	−0.139	0.076	3.316	0.870 (0.749–1.011)	0.069
DNA mismatch repair	−0.385	0.15	6.641	0.680 (0.507–0.912)	0.01
Perineural/vascular invasion	−0.026	0.09	0.08	0.975 (0.817–1.164)	0.777
**TNM stage**					
I					
II	0.238	0.137	2.999	1.268 (0.969–1.660)	0.083
III	0.59	0.137	18.505	1.803 (1.378–2.359)	< 0.001
**Differentiation**					
Well-differentiated					
Moderately differentiated	0.062	0.139	0.197	1.064 (0.810–1.396)	0.657
Poorly differentiated	0.207	0.093	20.71	1.231 (1.024–1.476)	< 0.001
**Site**					
Left-sided colon					
Right-sided colon	0.2662	0.106	2.491	1.305 (0.96–1.61)	0.062
Rectum	−0.05	0.09	0.551	0.951 (0.8–1.14)	0.581
BMI × Age	−0.044	0.037	−1.1831	0.96 (0.89–1.03)	0.236
BMI × Stage	0.026	0.044	0.609	1.03 (0.94–1.12)	0.542

### 3.3 Combined effects of BMI, age, and stage on risk of mortality

**Figure 2:**
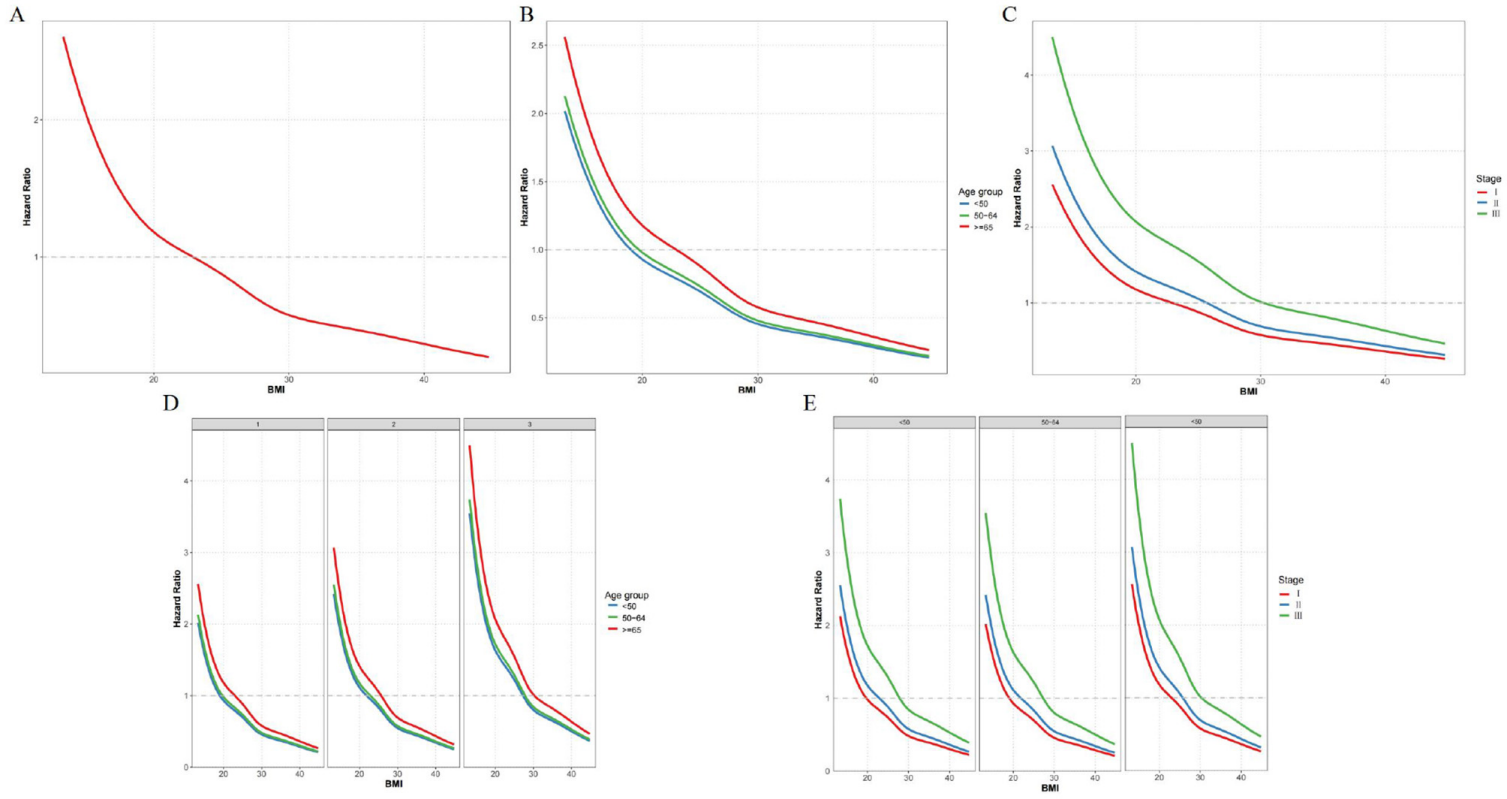
Combined effects of BMI, age, and stage on mortality risk. **(A)** BMI and mortality risk; **(B)** BMI and mortality risk by age group; **(C)** BMI and mortality risk by stage; **(D)** BMI and mortality risk by TNM stage stratified by age group; **(E)** BMI and mortality risk by age group stratified by TNM stage.

To evaluate the impact of BMI, age and stage on the risk of mortality in patients with colorectal cancer. Higher BMI is associated with lower mortality risk across the entire study population (Figure 2A). In patients aged 65 and older, the hazard ratio (HR) decreases sharply with increasing BMI, while in those younger than 50 and aged 50–64, the decline in HR is less steep (Figure 2B). In Stage III, there's a clear downward trend in HR with increasing BMI, Stage II shows a similar but less pronounced trend, and in Stage I, the HR decreases with higher BMI but the curve flattens at higher BMI values (Figure 2C).

Further stratified analysis by both age and stage reveals more detailed patterns. In Stage I, the decline in HR with increasing BMI is most pronounced in patients aged 65 and older, while younger patients (< 50) and those aged 50–64 show a less steep decline. In Stage II, all age groups show a moderate decline in HR with higher BMI, but the slope is steeper in the ≥65 group than in the younger groups. In Stage III, the decline in HR with increasing BMI is most pronounced in the oldest age group (≥65; Figure 2D). In Stage I, the HR decreases sharply with increasing BMI in patients aged 65 and older, while the decline is less steep in younger patients (< 50) and those aged 50–64. In Stage II, all age groups show a moderate decline in HR with higher BMI, but the slope is steeper in the ≥65 group than in the younger groups. In Stage III, the HR decreases with increasing BMI, but the curve flattens at higher BMI values across all age groups (Figure 2E).

### 3.4 Effects of BMI, age and stage on overall survival

**Figure 3:**
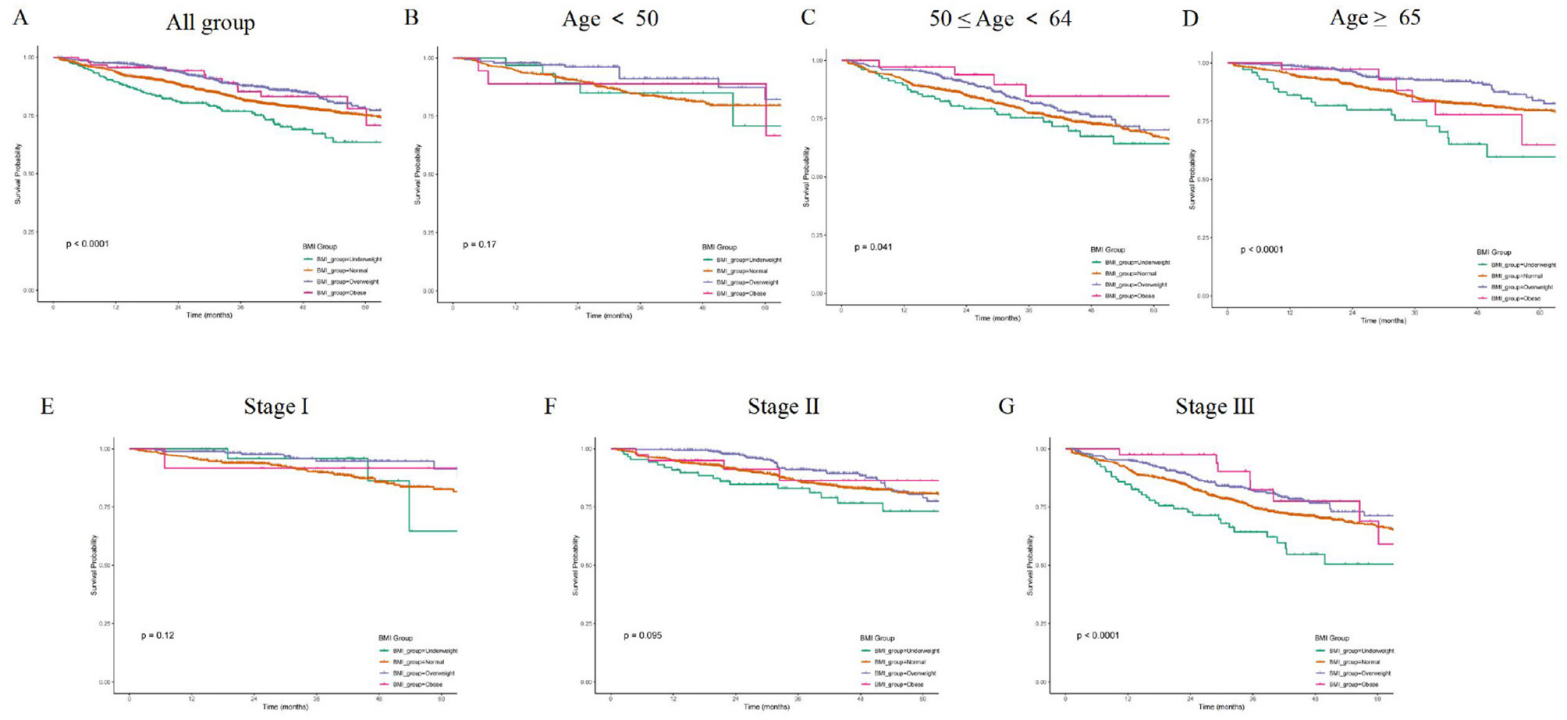
**(A)** K-M curves for overall survival across different BMI groups. **(B)** K-M curves for overall survival by BMI groups in patients aged < 50. **(C)** K-M curves for overall survival by BMI groups in patients aged 50–64. **(D)** K-M curves for overall survival by BMI groups in patients aged ≥65. **(E)** K-M curves for overall survival by BMI groups in Stage I patients. **(F)** K-M curves for overall survival by BMI groups in Stage II patients. **(G)** K-M curves for overall survival by BMI groups in Stage III patients.

The impact of BMI on overall survival in colorectal cancer patients varies across different age groups and TNM stages. Higher BMI is associated with better overall survival across the entire study population (*p* < 0.0001; Figure 3A). In patients younger than 50, there is no significant difference in overall survival between BMI groups (*p* = 0.17; Figure 3B). In the 50–64 age group, higher BMI is associated with significantly better overall survival (*p* = 0.041; Figure 3C). For those 65 and older, there is a significant association between higher BMI and better survival (*p* < 0.0001; Figure 3D). In Stage I patients, there is no significant difference in survival between BMI groups (*p* = 0.12; Figure 3E). Stage II patients show no significant difference in survival between BMI groups (*p* = 0.095; Figure 3F). In Stage III patients, higher BMI is associated with significantly better survival (*p* < 0.0001; Figure 3G).

## 4 Discussion

This study investigated the combined effects of BMI, age, and TNM stage on mortality risk in CRC patients. The primary findings were that higher BMI was associated with lower mortality risk across the entire study population, and this relationship was modified by age and TNM stage. Specifically, the protective effect of higher BMI was most pronounced in patients aged 65 and older, as well as in those with Stage III.

The relationship between BMI and CRC prognosis has been extensively studied, yet consensus remains elusive (14, 15). Our findings contribute to the ongoing debate about the “obesity paradox” in CRC. Some previous studies have reported similar protective effects of higher BMI on survival outcomes (10, 16, 17). For instance, a study by Jonathan et al. found that higher BMI was associated with better overall survival in CRC patients, particularly in those with Stage III disease (18). This is consistent with our results showing the most significant survival benefit in Stage III patients. Another study by Bette et al. also observed a survival advantage for overweight and obese CRC patients, suggesting that the obesity paradox might be a real phenomenon in specific patient populations (18, 19).

Conversely, other studies have reported conflicting results, indicating that higher BMI is associated with increased mortality risk in CRC patients (20). The inverse BMI-mortality association might partly reflect survivor bias—where a lower BMI captures individuals who experienced unintentional weight loss or who had previously lost weight because of occult disease—rather than a causal protective effect of adiposity itself. BMI also functions as a surrogate for fat-free mass and overall cardiorespiratory reserve, so the observed benefit could stem from preserved lean tissue rather than excess fat. Moreover, BMI cannot delineate body-fat percentage, visceral adipose distribution, or metabolic derangements, leading to misclassification of sarcopenic obesity and normal-weight obese phenotypes (21). This discrepancy might be due to differences in study populations, sample sizes, and methodological approaches (22). Our study's strength lies in its large sample size and comprehensive analysis of the interaction between BMI, age, and TNM stage, providing more nuanced insights into this complex relationship.

The mechanisms underlying the obesity paradox in CRC remain unclear, but several hypotheses have been proposed (14). One possible explanation is that higher BMI may be associated with a better nutritional reserve, which could help patients tolerate cancer treatments better and recover from complications more effectively (23, 24). Additionally, adipose tissue might have immunomodulatory effects that influence tumor biology and progression (25, 26). Furthermore, the protective effect of higher BMI could be related to differences in body composition, with higher muscle mass potentially contributing to better outcomes (27).

The modifying effects of age and TNM stage observed in our study might be attributed to biological and physiological differences across age groups and disease stages (28, 29). Older patients (≥65 years) with higher BMI might have a more favorable tumor biology and better prognosis (11). In advanced stages like Stage III, the impact of BMI on survival might be more pronounced due to the greater metabolic demands of the disease and the potential benefits of greater nutritional reserves (30, 31). This relationship was further supported by the analysis of overall survival. Higher BMI was associated with better overall survival across the entire study population (*p* < 0.0001). In patients aged 65 and older, there was a significant association between higher BMI and better survival (*p* < 0.0001). In Stage III patients, higher BMI was associated with significantly better survival (*p* < 0.0001). In our cohort, the relatively small proportion of Stage I cases among patients aged ≤ 50 years may be related to the distinct clinicopathological characteristics of early-onset colorectal cancer, which often presents with non-specific symptoms and exhibits more rapid progression, potentially leading to diagnosis at more advanced stages (13). In the absence of cancer-specific survival (CSS) and disease-free survival (DFS) data, our conclusions are based on all-cause death; future prospective studies with complete cause-of-death ascertainment and DFS data are warranted to determine whether the observed BMI survival benefit persists when CRC-related deaths are analyzed separately and when recurrence-free outcomes are considered.

The demographic characteristics of our study population revealed several notable patterns. Males constituted 59.6% of the study cohort, and the mean age at diagnosis was 61.1 years. These figures align with established epidemiological data indicating that CRC is more prevalent in males and typically diagnosed in older adults (32, 33). This study included CRC patients who had not received neoadjuvant chemoradiotherapy before surgery, as such treatment might influence patients' BMI (34, 35). The study also excluded patients with metabolic diseases and those using medications that could affect BMI, minimizing confounding factors related to BMI's influence on prognosis (36, 37). The proportion of male patients increased with rising BMI, a trend that has been previously documented and may reflect hormonal or metabolic differences between genders (38, 39). The lack of significant differences in other indicators across BMI groups suggests that BMI independently influences mortality risk, irrespective of factors like tumor size or lymph node involvement.

Our multivariate Cox regression analysis provided valuable insights into the relationships between key prognostic factors and mortality risk. Each unit increase in BMI was associated with a 7% decrease in mortality risk, reinforcing the obesity paradox. This finding persisted after adjusting for potential confounders such as age, sex, and cancer stage, indicating a robust association (17). Age emerged as a significant prognostic factor, with mortality risk increasing by 2.5% per year. This underscores the biological impact of aging on cancer progression and survival (40, 41). Tumor size and the number of positive lymph nodes were also strongly associated with mortality risk, highlighting their clinical relevance in risk stratification. Each additional harvested lymph node was associated with a 2.4% decrease in mortality risk, emphasizing the importance of thorough lymph node assessment during surgery (42, 43). The protective effect of dMMR status and the increased risk associated with poorly differentiated tumors further validate the biological significance of tumor genetics and histology in prognosis (44, 45). These findings align with existing knowledge and reinforce the need for comprehensive tumor characterization in clinical practice.

Despite the strengths of this study, including its large sample size and detailed analysis, several limitations should be acknowledged. First, as a retrospective study, it is subject to selection and information biases inherent in this design. Second, detailed information on body composition—such as skeletal muscle mass, visceral and subcutaneous fat distribution—was unavailable, which precluded us from distinguishing sarcopenic from non-sarcopenic obesity and may have obscured the true relationship between adiposity and survival. Third, patients who received neoadjuvant chemoradiotherapy were excluded because such treatment can acutely lower BMI, induce sarcopenia, and independently alter tumor biology and stage, thereby acting as a potential confounder; although this exclusion enhances internal validity, it limits the generalisability of our findings to contemporary populations in whom neoadjuvant therapy is standard. Fourth, data on lifestyle factors (physical activity, diet, smoking) were incomplete and may have introduced residual confounding. Future prospective cohorts that incorporate serial body-composition assessments and comprehensive treatment and lifestyle data in diverse populations are warranted to validate and extend these findings. Fifth, although our cohort was drawn from two high-volume tertiary centers, the study remains restricted to patients who underwent curative-intent surgery for Stage I–III disease. Consequently, non-surgical and metastatic (Stage IV) CRC cases were not represented, which may limit the generalizability of our findings to the broader CRC population.

This study highlights the complex interplay between BMI, age, and TNM stage in influencing mortality risk in CRC patients. The findings suggest that the obesity paradox is not uniform across all patient subgroups and should be interpreted within the context of individual patient characteristics. These results can help inform clinical decision-making and risk stratification, ultimately improving the prognosis and management of CRC patients.

## 5 Conclusions

Higher BMI is associated with lower mortality risk in colorectal cancer patients, particularly in those aged 65 and older and those with Stage III disease. These findings highlight the importance of considering BMI, age, and TNM stage jointly in clinical practice for CRC patients.

## Data Availability

The raw data supporting the conclusions of this article will be made available by the authors, without undue reservation.
